# Assessment of Blood Pressure Control Status Among Hypertensive Patients Attending Rwandan District Hospital NCD Clinics: A Retrospective Follow-Up Study

**DOI:** 10.1007/s44197-025-00356-3

**Published:** 2025-02-03

**Authors:** Evariste Ntaganda, Ziad El-Khatib, Regine Mugeni, Boniface Nsengiyumva, Clarisse Musanabaganwa, James Gafirita, Francois Uwinkindi, Richard Kalisa

**Affiliations:** 1 School of Public Health, College of Medicine and Health Sciences, University of Rwanda, Kigali, Rwanda; 2 Rwanda Biomedical Center, Ministry of Health, Kigali, Rwanda; 3 Department of Global Public Health, Karolinska Institutet, Stockholm, Sweden; 4 Kibagabaga District Hospital, Kigali, Rwanda; 5 School of Medicine and Pharmacy, College of Medicine and Health Sciences, University of Rwanda, Kigali, Rwanda

**Keywords:** Antihypertensive, Blood pressure control, Cardiovascular diseases, Hypertension, NCD clinics

## Abstract

**Background:**

Hypertension is a major public health issue and a leading risk factor for cardiovascular disease (CVD). We assessed blood pressure (BP) control among adult hypertensive patients attending non-communicable disease (NCD) clinics in five Rwandan district hospitals.

**Methods:**

We extracted data on hypertensive management from five Rwandan district hospitals from June 2016 to August 2021. BP control was defined as systolic blood pressure (SBP) < 140 mmHg and diastolic blood pressure (DBP) < 90 mmHg measured within the last four months. We performed statistical analysis using chi-square tests and multivariate regression analyses with 95% confidence intervals (CI).

**Results:**

Blood pressure control was achieved in 41.5% of hypertensive patients (*n* = 438/1,055). The majority were aged > 60 years (mean age 62; *n* = 663/1,055; 62.8%), and women, with approximately three-quarters of patients (*n* = 796/1,055; 75.5%) had a BMI between 18.5 and 24.9 Kg/m^2^ and the majority (*n* = 843/1,055; 79.9%) resided in rural districts. More than half (*n* = 585/1,055; 55.5%) were taking two antihypertensive medications. Factors significantly associated with uncontrolled BP included BMI *≥* 30 kg/m^2^ (*p* < 0.001), use of Angiotensin-converting enzyme (ACE) inhibitors (*p* = 0.01), use of four antihypertensive drugs (*p* = 0.013), and missing an NCD clinic appointment (*p* < 0.001).

**Conclusions:**

BP control rates among hypertensive patients attending NCD clinics remain low. Strengthening patient counseling, encouraging physical activity, and improving medication adherence are critical. Building the capacity of healthcare staff at both hospital and health centre levels is vital to improving hypertension management in NCD clinics.

**Supplementary Information:**

The online version contains supplementary material available at 10.1007/s44197-025-00356-3.

## Introduction

Hypertension (HTN) is a major public health issue and a leading risk factor for cardiovascular disease (CVD). Globally, approximately 17.9 million deaths are attributed to CVD annually, with low- and middle-income countries (LMIC) accounting for nearly 80% of these deaths [1, 2]. By 2030, CVD mortality is projected to double, with a particularly significant impact in sub-Saharan Africa (SSA) [2]. HTN is the leading risk factor for CVD mortality, accounting for 45% of heart disease deaths and 51% of stroke deaths due to uncontrolled blood pressure (BP) [3]. In certain age groups, CVD risk doubles with every 20 mmHg increase in systolic blood pressure (SBP) and 10 mmHg increase in diastolic blood pressure (DBP). To reduce deaths associated with CVD complications such as stroke, myocardial infarction, and renal disease, optimal BP control is defined as maintaining SBP and DBP below 140/90 mmHg [3–5]. In addition to lifestyle changes, urbanization, behavioral habits, dietary practices, and genetics contribute to uncontrolled BP and require targeted interventions [6, 7].

Advancements in BP measurement and the availability of antihypertensive medications have improved hypertension management. However, many patients fail to achieve adequate BP control, leading to high morbidity and mortality from hypertension-related complications [5, 7]. In high-income countries, less than 27% of patients with HTN have controlled BP, and in LMIC, fewer than 10% have achieved control [2, 8]. In Rwanda, limited studies on HTN control exist, but an estimated 62% of hypertensive patients have uncontrolled BP [9]. Without proper follow-up, uncontrolled BP poses a significant risk of complications and death, even after treatment initiation. The primary goal of treatment is to maintain BP within controlled ranges [10]. Uncontrolled BP significantly increases morbidity, causing conditions such as stroke, heart attack, and kidney disease, and resulting in frequent consultations and prolonged hospitalizations [11].

Globally, the detection, treatment, and control of HTN remain inadequate [12]. Limited knowledge exists about how hypertensive patients manage their BP and the factors contributing to poor BP control. Research indicates a range of factors associated with uncontrolled BP, including age, gender, occupation, smoking status, duration since HTN diagnosis, poor medication adherence, lack of exercise, high salt intake, being overweight or obese, physician inertia, inability to afford medication, presence of comorbidities, and alcohol consumption [9–13].

Preventing HTN complications requires identifying BP control challenges and implementing effective solutions [2, 5, 7]. Understanding the prevalence of uncontrolled BP among hypertensive patients can guide healthcare providers in improving HTN management and help policymakers develop context-specific interventions. We aimed to assess BP control status among adult hypertensive patients attending NCD clinics at Rwandan district hospitals.

## Methods

### Study Design and Settings

This retrospective cohort study was conducted among adult patients (≥ 18 years) with HTN enrolled in NCD clinics at five district hospitals (DH) between June 2016 and August 2021. These five hospitals, each representing one of Rwanda’s five provinces, collectively serve a population of 2.3 million [14].

Since 2016, the Rwanda ministry of health has introduced NCD clinics at hospitals and health centers to treat CVD, diabetes, chronic respiratory diseases, and HTN. These clinics are managed by nurses and medical officers. Attending NCD nurses and medical officers received clinical mentorship and supportive supervision from implementing partners and the Rwanda Biomedical Center (RBC) to ensure retention, motivation, and skill development [15, 16], as well as to ensure quality care aligned with national NCD treatment guidelines [17]. To increase the detection rate, community health workers encourage people to undergo screening by nurses, and individuals identified with hypertension are referred to nearby health centers or hospitals for management [17]. While only 3% of the population has private health insurance, the majority rely on community-based health insurance, which requires an annual contribution of RWF 3,000 (approximately US$3) and a 10% co-payment per illness episode. In case of medicine shortage, patients must procure unavailable medications from private pharmacies [14].

### Inclusion and Exclusion Criteria

All hypertensive patients aged *≥* 18 years enrolled in the five hospital NCD clinics during the study period were included. Newly diagnosed patients (*≤* 12 months) enrolled in the routine NCD follow-up clinic program were excluded.

### Sample Size Estimation and Sampling

Sample size was calculated using a standard formula for proportions to estimate the number of participants for the study.

n = Z2 P (1-P)/e2 where: n = Sample size; z = level of confidence; p = estimated prevalence; e = margin of error.

In the absence of similar studies in Rwanda, a prevalence rate of 50% (95% CI) and a margin of error of 3% were used for sample size calculation. From a population of 9,104 participants across the five district hospitals, the estimated sample size for this study was calculated. The number of participants from the five selected hospitals was as follows: Gisenyi (west), Kirehe (East), Masaka (Kigali), Munini (South) and Nemba (North) with 176, 205, 217, 225, and 236 participants, respectively.

Given the estimated prevalence *p* = 0.50, the 95% confidence interval (Z = 1.96), and margin of error of 3%, the estimated sample size was *n* = 1.962 × 0.5 (1-0.5)/0.032 = 956. To account for potential missing participant files due to the retrospective nature of the study, 99 additional files (10%) were included, resulting in a minimum sample size of 1,055 participants. Systematic sampling was used to ensure a broad representative of data extracted from the EMR until the required sample size was achieved.

### Data Collection Procedure and Quality Control

Trained nurses, supervised by the principal investigator, extracted data from the EMR of the five selected hospitals between May and August 2022. Information collected included socio-demographic, disease-related, and medication-related factors as detailed below. To ensure data quality and consistency, research assistants received prior training on study procedures and data extraction. Extracted data were checked for completeness and consistency during management, storage, and analysis.

### Study Variables

#### Independent Variables

(a) Socio‑demographic variables (gender, age, residence, marital status, and hospital attended); (b) Disease‑related factors (body mass index (BMI), presence of comorbidities, stage of hypertension (stages 1, 2, and 3) at enrolment, as defined somewhere else [6], frequency of follow-up, BP measurement, and ever missed appointments); and (c) Medication‑related factors (Duration of anti-hypertensive treatment, adherence to anti-hypertensive treatment, treatment modification, class of anti-hypertensive drugs, and number of medications).

#### Dependent Variable

BP control status (controlled vs. uncontrolled) during the last four months of NCD clinic follow-up. BP control was defined as having SBP of < 140 mmHg and DBP < 90 mmHg in four consecutive months period from the time of study enrollment. For this study, subsequent appointments were scheduled every one to three months based on patients’ BP control status, with a maximum interval of four months.

### Statistical Analysis

Data extracted from the EMRs were exported to Ms. Excel for cross-checking to identify and remove potential errors. Exploratory data analysis was conducted to identify outliers and assess data skewness, and to determine appropriate measures of central tendency. Descriptive statistics, as well as bivariate and multivariate analyses, were used to describe the socio-demographic, disease-related, and medication-related characteristics of the study population.

Bivariate analysis examined the relationship between independent variables and BP control status (adequate vs. inadequate). Pearson´s Chi square test or Fisher´s exact test was used to compare categorical variables between controlled and uncontrolled BP groups. The results of the bivariate analysis informed the multivariable analyses. Statistically significant predictor variables from the bivariate analysis were included in a multivariate analysis using a stepwise backward logistic regression model. This adjusted for potential confounders and identified factors associated with adequate BP control. Associations between predictor and outcome variables were estimated using p-values, 95% confidence interval (CI), crude odds ratios (COR), and adjusted odds ratios (AOR). A significance threadshold of *p* < 0.05 was used. Analyses were performed using Stata version 15.

### Study Approval

This study received approval from the Rwanda National Ethics committee (RNEC) (Approval ID# 699/RNEC/2021). All data were obtained from the hospital EMR system, which was developed by the NCD division within the Rwanda Biomedical Center (RBC) - an implementation body under the Rwanda Ministry of Health responsible for delivering health services nationwide.

### Consent for Publication

This study utilized routinely collected data from the NCD division’s programmatic activities, with approval granted by the NCD Division (Reference ID# 13251/RBC/NCD/21). As this was a secondary analysis of de-identified data collected as part of a national program, individual participant consent was not required.

## Results

### Socio‑Demographic Characteristics

**Fig. 1:**
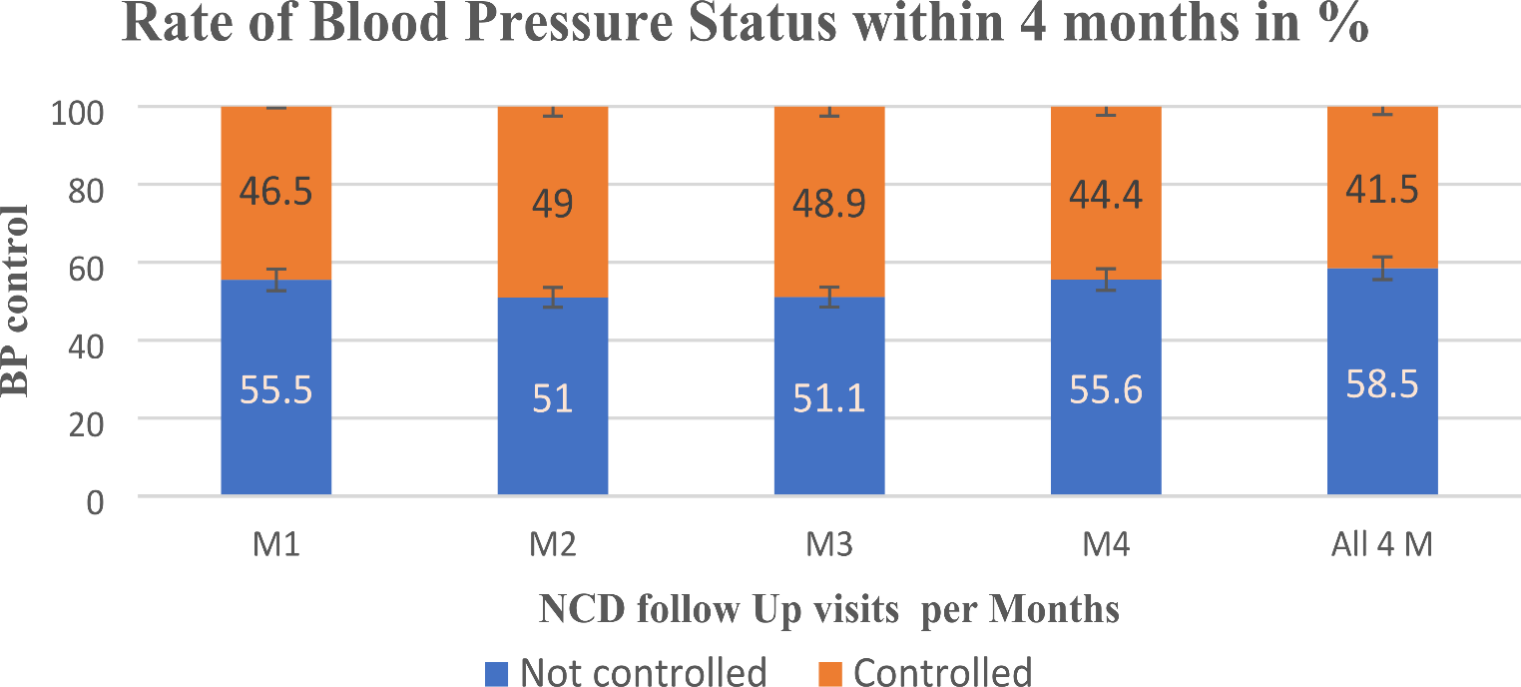
Blood pressure control status among hypertensive patients

A total of 438/1,055 (41.5%) of the patients had achieved BP control (Fig. 1). The majority of patients (663; 62.8%) were > 60 years of age (mean age: 62; 62.8%, *n* = 663/1,055). Most women were women (75.5%; *n* = 796/1,055), resided in rural districts (79.9%; *n* = 843/1,055), resided in rural districts (79.9%; *n* = 843/1,055), and had a BMI between 18.5 and 24.9 Kg/m^2^ (Table 1).

**Table 1:** Socio‑demographic characteristics of hypertensive patients attending hospital NCD follow-up clinics

Variable	Frequency (%) *n*/*N*
Sex	
Female	796 (75.5%)
Male	259 (24.6%)
Age (Years)	
Mean ± SD	62 ± 14
< 40 years	79 (7.5%)
40–59 years	313 (29.7%)
$\:\ge\:60$ years	663 (62.8%)
BMI category (Kg/m^2^)	
< 18.5	78 (8.4%)
18.5–24.9	514 (55.5%)
25-29.9	206 (22.2%)
> 30	129 (13.9%)
Missing	128 (12.1%)
Residence	
Rural	843 (79.9%)
Urban	212 (20.1%)
Hospital NCD clinics (Province)	
Gisenyi (West)	225 (21.3%)
Kirehe (East)	232 (22.0%)
Masaka (Kigali)	205 (19.4%)
Munini (South)	176 (16.7%)
Nemba (North)	217 (20.6%)

### Co-Morbidities and Patients’ Follow-Up Visits

Out of 1,055 patients, less than half (*n* = 435; 41.2%) were initially enrolled in the NCD clinic with stage 2 hypertension. These patients attended monthly follow-up visits, with a median follow-up duration of two years. Kidney disease was reported as a comorbid condition in 10% of patients (*n* = 105). Additionally, 37.8% (*n* = 397) missed at least one appointment, and 66.3% (*n* = 699) received treatment at their local health center’s NCD clinic (Table 2).

**Table 2:** Co-morbidities and patients follow-up visits

Variable	*N* (%)
Hypertension stage at enrollment	
Stage 1	328 (31.1%)
Stage 2	435 (41.2%)
Stage 3	292 (27.7%)
Kidney diseases	
No	1,028 (97.4%)
Yes	10 (0.9%)
Missing	17(1.6%)
Diabetes	
No	957 (90.7%)
Yes	81 (7.7%)
Missing	17(1.6%)
Heart diseases	
No	1,022 (98.5%)
Yes	16 (1.5%)
Missing	17(1.6%)
Follow-up status	
Transferred to Health center	699 (66.3%)
Lost to follow up	229 (21.7%)
Died	11 (1.0%)
Still in follow up at hospital	116 (11.0%)
Ever missed appointment	
No	654 (62.2%)
Yes	397 (37.8%)
Missing	104 (9.9%)

### Medication Related Characteristics

Thiazide diuretics (82.4%; *n* = 869) and calcium channel blockers (60.8%; *n* = 642) were the most commonly prescribed single antihypertensive medications. However, over half of the patients (*n* = 585; 55.5%) did not achieve BP control (Table 3).

**Table 3:** Type of antihypertensives medicine used

Variable	*N* (%)
Hydrochlorothiazide	
No	186 (17.6%)
Yes	869 (82.4%)
ACEIs	
No	806 (76.4%)
Yes	249 (23.6%)
Calcium channel blockers (Nifedipine &Amlodipine)	
No	413 (39.2%)
Yes	642 (60.8%)
Beta blockers (Atenolol)	
No	994 (94.2%)
Yes	61 (5.8%)
Alpha blockers (Methyl dopa)	
No	1,010 (95.7%)
Yes	45 (4.3%)
Angiotensin receptor blockers	
No	1,048 (99.3%)
Yes	7 (0.7%)
Drug combination (feel-pills)	
Monotherapy	327 (31.0%)
Bitherapy	585 (55.5%)
Tripletherapy	111 (10.5%)
Quadritherapy	10 (0.9%)

### Status of Blood Pressure Control

BP control, defined by systolic and diastolic BP outcomes, was achieved in 41.5% of patients (*n* = 438/1,055). The remaining 58.5% of patients did not achieve BP control, defined as BP < 140/90 mmHg sustained over four consecutive months (Fig. 1).

### M: Month Factors Associated with Uncontrolled Blood Pressure

In the bivariate analysis, patients taking single medication - either angiotensin-converting enzyme (ACE) inhibitors, calcium channel blockers, or a drug combination– those who had ever missed an appointment, and the stage of hypertension at enrollment (*p* = 0.005) were statistically associated with uncontrolled BP (Supplementary Table 1). After adjusting for potential confounding factors using multivariable binary logistic regression analysis, the following factors were significantly associated with poor hypertension control: BMI > 30 kg/m^2^ (*p* < 0.001), use of ACE inhibitors (*p* = 0.01), combination therapy with four antihypertensive drugs (*p* = 0.013), and missing an appointment (*p* < 0.001) (Table 4).

**Table 4:** Factors associated with uncontrolled blood pressure among hypertensive at hospital NCD follow-up clinics

Variable	Full model				Reduced model			
	OR	95% CI		p-value	OR	95% CI		p-value
BMI of *≥* 30 (Kg/m^2^)	1.06	0.69	1.64	0.786	0.68	0.48	0.95	< 0.001
Hospital NCD clinics (Province)								
*Kirehe (East)*	0.36	0.22	0.59	< 0.001				
*Masaka (Kigali)*	0.58	0.35	0.97	0.036	0.35	0.24	0.53	*P* < 0.001
*Munini (South)*	1.68	1.03	2.76	0.039	0.56	0.38	0.82	0.003
*Nemba (North)*	1.10	0.67	1.80	0.7	1.65	1.11	2.47	0.014
Taking ACE inhibitors	0.65	0.42	1.01	0.054	0.63	0.44	0.90	0.01
Four medicines(feel-pills)	1.92	0.39	9.51	0.424	0.69	0.52	0.92	0.013
Missed appointment	0.48	0.32	0.70	< 0.001	0.49	0.36	0.67	< 0.001

## Discussion

Our findings revealed that only 41.5% of hypertensive patients attending Rwandan district hospitals had controlled BP, despite receiving long-term treatment. Studies conducted in Ethiopia reported similar control rates of 41.2% [18]. However, our findings were slightly higher than the 33% control rates reported in other [19]. Studies conducted in Uganda, Kenya, and East and West African countries found control rates of 46% and 47.3%, respectively, which are slightly higher than our findings [20, 21]. Other studies from the USA reported higher BP controlled rates, ranging from 51.1 to 73.4% (22). These differences may be attributed to sociocultural factors, patient behavior, healthcare professional expertise, and variations in healthcare services across different study settings [21, 22]. Additionally, inconsistencies may also be linked to differences in criteria used to classify hypertension control and antihypertensive drug adherence rates. Our study followed the Seventh Joint National Committee (JNC7) guidelines, which were adapted into the national NCD management guidelines [6, 17].

Some authors have reported hypertension control rates in high-income countries that are still < 50% [22], which is comparable to our study findings. This reflects improvements in hypertension management in Rwanda, driven by the decentralization of NCD clinics and strengthened nurse-to-nurse mentorship programs. These programs have ensured adequate care for approximately two-thirds of hypertensive patients [22, 23]. While we acknowledge that this study did not include potentially significant variables such as medication dispensing and prescription patterns, our findings suggest that the decentralized NCD clinic model has enhanced access to hypertension management and improved patient retention. This was reinforced by measures including early detection, diagnosis, and treatment of hypertension, especially in rural populations, coupled with opportunistic screening campaigns to mitigate barriers like low health literacy, inadequate treatment adherence, and systematic health policy [23–25].

Obese patients in our study had significantly lowers odds of achieving BP control (OR = 0.68, 95%CI 0.48–0.95, *p* < 0.001) over four consecutive clinic visits, compared to those with normal BMI. Similar studies have reported that overweight and obese individuals have higher odds of uncontrolled BP (OR 1.73, 95%CI 1.15–2.58, *p* < 0.001) [26, 27]. Obesity is known to induce resistance related to adipose tissue, affecting the renin-angiotensin system and leading to increased intra-abdominal and intravascular fat, sodium retention, and sympathetic nervous system activation, all contributing to obesity-related hypertension [28, 29]. National efforts to reduce overweight and obesity through physical activity and healthy eating should be prioritized. Health promotion campaigns encouraging mass participation in sports could help reverse the growing burden of NCD [30].

Missing clinic appointments was statistically associated with uncontrolled hypertension (OR = 0.49, 95% CI 0.36–0.67, *p* < 0.001). This finding aligns with a study conducted in Ghana, where missed clinic appointments were similarly associated with uncontrolled BP (OR = 0.43, 95% CI 0.22–0.86, *p* < 0.018). Common reasons for missed appointments included lack of transportation, financial constraints, and forgetfulness [31]. Other studies identified additional reasons, including perceived clinical stability, work commitments, and long waiting times at health facilities [32]. Forgetting appointments was often attributed to low awareness of the impact of missed treatment. Implementing flexible appointment scheduling could help address this issue.

Our findings showed that 55.5% of patients were on two-drug therapy, while patients on a four-drug regimen had significantly higher rates of uncontrolled BP (OR = 0.49, 95%CI 0.36–0.67, *p* < 0.001) during the study period. This is consistent with other studies linking multidrug regimens to poor BP control (OR = 0.41, 95%CI 0.26–0.64, *p* = 0.013). Patients on *≥* 3 medications were 2.4 times less likely to have their BP control compared to those on fewer medications. This suggests that patients with more severe hypertension may face greater challenges in achieving BP control, prompting healthcare providers to employ more aggressive drug regimens [32]. Thus, fixed-dose combinations should be prioritized for inclusion on the national essential medicines list to improve adherence, affordability, effectiveness, and convenience in BP control [7, 33, 34].

This study found that the use of ACE inhibitors was associated with lower BP control. This was expected, as the recommended first-line treatment for Black populations with hypertension is calcium channel blockers. Thiazide diuretic and ACE inhibitors were negatively associated with BP control compared to calcium channel blockers [35, 36]. This trend was also observed in our study, where ACE inhibitors/ARB were among the most commonly prescribed drugs, which may partly explain the lower rates of BP control. Further investigation is needed to explore potential biases. Other studies have identified a range of risk factors for uncontrolled BP, including advanced age, obesity, high sodium intake, diabetes, and kidney disease [7, 24, 29, 36].

A key strength of this study was its examination of HTN management across one district hospital in each Rwandan province. We believe these results are representative, as the socio-demographic characteristics and access to care are comparable across district hospitals in Rwanda [14]. However, this study has some limitations: Its retrospective design, which relied on routinely collected medical data, introduces potential bias due to missing information in the EMR. This could have led to over- or under-estimation of uncontrolled BP rates. Additionally, the study design did not allow for the determination of temporal or causal relationships between dependent and predictor variables.

We could not ascertain the specific reasons for low BP control rates, but we described the magnitude of the problem and highlighted gaps in documentation. While it is difficult to conclude whether specific tests were omitted, the documentation clearly demonstrates room for improvement in patient care. The evaluation of hypertensive patients should be comprehensive, including the identification of risk factors, comorbidities, and patient stratification for appropriate treatment. NCD clinics provide an opportunity to improve hypertension management and prevent adverse outcomes associated with poor BP control.

## Conclusion

The proportion of hypertensive patients with uncontrolled BP was relatively high in Rwandan district hospital NCD clinics. A BMI of *≥* 30 kg/m^2^, the use of ACE inhibitors, combination therapy with four antihypertensive drugs, and having ever missed an appointmentt were significantly associated with uncontrolled BP. Strengthening patient counselling on physical activity and medication adherence in CD clinics is essential. Therefore, we recommend capacity building to all levels of healthcare staff to improve hypertension management in NCD clinics at health centres, as well as ensuring the availability of calcium channel blockers as the recommended first-line treatment. Further research should investigate barriers to effective BP control and develop targeted interventions to improve treatment outcomes.

## Electronic supplementary material

Below is the link to the electronic supplementary material.

Supplementary Material 1

## Data Availability

No datasets were generated or analysed during the current study.

## Abbreviations

ACEI: Angiotensin converting enzyme inhibitors
AOR: Adjusted odds ratio
BMI: Body mass index
BP: Blood pressure
CBI: Community based insurance
CCB: Calcium Chanel Blockers
CI: Confidence interval
COR: Crude odds ratio
CVD: Cardiovascular diseases
DBP: Diastolic Blood pressure
DH: District Hospital
EMR: Electronic medical Record
HC: Health Centers
HTN: Hypertension
NCD: Non communicable diseases
OR: Odds ratio
SBP: Systolic blood pressure
SSA: Sub Sahara Africa
WHO: World Health Organization
PEN: Package of essential interventions for non-communicable disease
RBC: Rwanda Biomedical Center
