# Supplementary material for: Assessment of Blood Pressure Control Status Among Hypertensive Patients Attending Rwandan District Hospital NCD Clinics: A Retrospective Follow-Up Study
Source: J Epidemiol Glob Health. 2025 Feb 3;15(1):13. doi: 10.1007/s44197-025-00356-3 (PMC11790538; doi:10.1007/s44197-025-00356-3)
Supplement: Supplementary file 1 — Supplementary Material 1 [file 44197_2025_356_MOESM1_ESM.docx]

**Supplementary Table 1**: Bivariate analysis of the likelihood of hypertension control among patients attending hospital NCD follow-up clinics

| Variable | Not controlled | Controlled | p-value |
| --- | --- | --- | --- |
| Hypertension status (n=1055) | 617 (58.5%) | 438 (41.5%) |  |
| Sex |  |  | 0.427 |
| Female | 471 (59.2%) | 325 (40.8%) |  |
| Male | 146 (56.4%) | 113 (43.6%) |  |
| Age (years) |  |  | 0.068 |
| <40 | 55 (69.6%) | 24 (30.4%) |  |
| 40-59 | 173 (55.3%) | 140 (44.7%) |  |
| ≥60 | 389 (58.6%) | 274 (41.3%) |  |
| BMI category(n=927) |  |  | 0.090 |
| <18.5 | 45 (57.7%) | 33 (42.3%) |  |
| 18.5-24.9 | 289 (56.2%) | 225 (43.8%) |  |
| 25-29.9 | 137 (66.5%) | 69 (33.5%) |  |
| ≥30 | 76 (58.9%) | 41 (42.7%) |  |
| Residence |  |  | 0.160 |
| Rural | 484 (57.4%) | 359 (42.6%) |  |
| Urban | 133 (62.7%) | 79 (37.3%) |  |
| Follow up hospital |  |  | <0.001 |
| Gisenyi (West) | 130 (57.8%) | 95 (42.2%) |  |
| Kirehe (East) | 170 (73.3%) | 62 (26.7%) |  |
| Masaka (Kigali) | 143 (69.8%) | 62 (30.2%) |  |
| Munini (South) | 61 (34.6%) | 115 (65.4%) |  |
| Nemba (North) | 113 (52.1%) | 104 (47.9%) |  |
| Hydrochlorothiazide |  |  | 0.308 |
| No | 115 (61.8%) | 71 (38.2%) |  |
| Yes | 502 (57.8%) | 367 (42.2%) |  |
| ACEIs |  |  | <0.001 |
| No | 442 (54.8%) | 364 (45.2%) |  |
| Yes | 175 (70.3%) | 74 (29.7%) |  |
| Calcium channel blockers (Nifedipine, Amlodipine) |  |  | <0.001 |
| No | 225 (54.5%) | 188 (45.5%) |  |
| Yes | 392 (70.3%) | 250 (38.9%) |  |
| Beta blockers: Atenolol |  |  | 0.247 |
| No | 577 (58.1%) | 417 (41.9%) |  |
| Yes | 40 (65.6%) | 21 (34.4%) |  |
| Alpha blockers: Methyldopa |  |  | 0.148 |
| No | 586 (58.0%) | 424 (42.0%) |  |
| Yes | 31 (68.9%) | 14 (31.1%) |  |
| ARBs: Valsartan, losartan and candesartan |  |  | 0.613 |
| No | 613 (58.5%) | 435 (41.5%) |  |
| Yes | 4 (57.1%) | 3 (42.9%) |  |
| Number of drugs |  |  | <0.001 |
| One drug | 164 (49.7%) | 166 (50.3%) |  |
| Two drugs | 361 (60.8%) | 247 (39.2%) |  |
| Three drugs | 84 (70.6%) | 35 (29.4%) |  |
| Four drugs | 7 (63.6%) | 4 (36.4%) |  |
| Follow-up status |  |  | <0.001 |
| Transferred to Health Centre | 376 (53.8%) | 323 (46.2%) |  |
| Lost to follow up | 163 (71.2%) | 66 (28.8%) |  |
| Died | 5 (45.4%) | 6 (54.5%) |  |
| Still in follow up at hospital | 73 (62.9%) | 43 (37.1%) |  |
| Ever missed appointment |  |  | <0.001 |
| No | 334(51.1) | 320(48.9) |  |
| Yes | 282(71) | 115(29) |  |
| Hypertension stage enrollment |  |  | 0.005 |
| Stage 1 | 168(51.22) | 160(48.78) |  |
| Stage 2 | 266(61.15) | 169(38.85) |  |
| Stage 3 | 183(62.67) | 109(37.33) |  |
| Comorbidities |  |  |  |
| Kidney disease |  |  | 0.770 |
| No | 611 (58.5%) | 433 (41.5%) |  |
| Yes | 6 (54.5%) | 5 (45.4%) |  |
| Diabetes |  |  | 0.189 |
| No | 564 (57.9%) | 410 (42.1%) |  |
| Yes | 53 (65.4%) | 28 (34.5%) |  |
| Heart disease |  |  | 0.611 |
| No | 609 (58.5%) | 430 (41.5%) |  |
| Yes | 8 (50.0%) | 8 (50.0%) |  |

* Fisher exact test
